# Damage Monitoring and Localization Imaging of Aluminum Alloy Thin-Walled Structure Based on Remote Bonding Fiber Bragg Gratings Sensing

**DOI:** 10.3390/ma17030652

**Published:** 2024-01-29

**Authors:** Lu Han, Mi Wang, Lindong Chai, Dingyun Liu, Weifang Zhang, Wei Zhang

**Affiliations:** 1 School of Reliability and Systems Engineering, Beihang University, Beijing 100191, China; hanlu13051827622@buaa.edu.cn (L.H.); wangmi0526@buaa.edu.cn (M.W.); chailindong@buaa.edu.cn (L.C.); 08590@buaa.edu.cn (W.Z.); 2 Nanjing Chenguang Group Co., Ltd., Aerospace Servo Technology Research Institute, Nanjing 210006, China; liudingyun1914@buaa.edu

**Keywords:** Structural Health Monitoring (SHM), remote bonding, damage index, Lamb wave, signal denoising processing

## Abstract

In this paper, the damage monitoring investigation based on the remote bonding fiber Bragg grating sensing is performed on the aerospace aluminum alloy thin-walled structure with prefabricated damage. Firstly, an ultrasonic excitation-fiber Bragg gratings (UE-FBGs) sensing experimental platform is established for the simulation of defects monitoring, in which the sensors are placed at a certain distance from the bonding area. Secondly, different arrangements of exciters and receivers are utilized for the original signals and the damage signals. Subsequently, the raw signals are processed by filter and feature extraction in order to denoise the signals and acquire the parameters sensitive to the damage. Finally, an improved Reconstruction for Image Defects (RAPID) algorithm is used to locate and reconstruct the pre-existing damage. The results show that the proposed system improves the sensitivity of the FBG receiver signal and the accuracy of the damage imaging.

## 1. Introduction

Structural Health Monitoring (SHM) is an emerging technology that employs advanced sensor equipment to acquire and analyze signals for obtaining structural health-related information. By integrating knowledge of structural mechanics and material failure, SHM provides a comprehensive diagnosis of a structure’s health status [1,2]. SHM has been widely applied in fields such as aerospace [3], rail transportation [4], and new energy batteries [5]. SHM currently relies on various monitoring techniques, such as ultrasound wave-based [6], strain-based [7], temperature-based [8], and electromechanical impedance-based [9] methods.

Ultrasonic Lamb wave-based monitoring is widely used for rapid damage detection of structures made of metallic materials and composites [6,10]. Because Lamb waves have the advantages of long propagation distance, fast detection, and the ability to propagate within the plate, Lamb wave-based monitoring is suitable for damage monitoring of large thin-walled structures. Piezoelectric (PZT) transducer and fiber optic grating (FBG) sensors are the two most commonly used types of sensors in Lamb wave monitoring [11]. PZT transducers are capable of transmitting and receiving Lamb wave signals. Compared to ultrasound excitation (UE) devices, the PZT transducer is smaller and lighter and can monitor a larger area [12]. FBG sensors are small in size, lightweight, resistant to electromagnetic interference, and can be multiplexed [13]. The UE- FBGs damage detection techniques is a newly developed SHM technology that generates ultrasonic Lamb waves using PZT sensors and receives them using FBG sensors [14]. This technology has the advantages of both PZT and FBG and has good application prospects in aircraft that have high requirements for the monitoring system’s volume and weight. Therefore, this paper presents a quantitative assessment of damage localization imaging of aerospace aluminum alloy plates based on UE- FBGs.

Compared to measuring static strain in structures, detecting ultrasound waves using FBG requires higher sensitivity and wider bandwidth to ensure clear waveform detection of ultrasonic Lamb waves in plate-like structures. Therefore, meticulous design of fiber optic grating sensors and their corresponding demodulation systems is crucial. Currently, there are several commonly used high-speed demodulation methods, including edge filtering demodulation using a broadband light source and power demodulation using a laser source [15]. Edge filtering demodulation technology uses a broadband light source to emit light, and a photodetector detects changes in the reflected spectrum intensity of the FBG to perceive variations in wavelength. Perez et al. [16] proposed a matched filtering demodulation system that successfully detected signals generated by a PZT transducer using two fiber optic gratings with slightly different Bragg wavelengths. However, the use of a broadband light source may reduce the effective resolution of the system due to low light source intensity and high noise in the signal. Power demodulation technology employs a high-power narrowband laser source for optical signal excitation, and the reflected wavelength is solved by measuring the output light intensity through a photodetector, which has the advantages of high sensitivity and good signal-to-noise ratio (SNR). Tsuda et al. [17] used a tunable laser source to construct an FBG-based ultrasonic sensing system. Jiang et al. [18] emitted narrowband laser light through a tunable laser source and performed power demodulation. The results showed that when the length of the grating was much smaller than the ultrasonic wavelength, the ultrasonic wave could be effectively detected. This study analyzes the process of changes in ultrasonic signals at the site of structural damage. Based on the principle of power demodulation, an experimental setup for ultrasonic excitation-FBG sensing damage monitoring is built.

**Figure 1:**
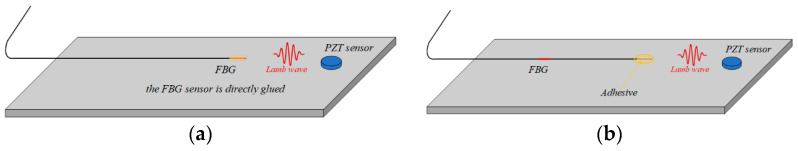
The schematic diagram of remote bonding [19]. (**a**) Directly bonding; (**b**) remote bonding.

To address the issue of low amplitude of ultrasonic signals received by FBGs, it is possible to enhance the efficiency of transferring the strain caused by ultrasonic signals to the FBGs. One method is to use a remote bonding approach to convert the mode of ultrasonic signals and increase their amplitude. The schematic diagram of remote bonding is shown in the Figure 1:

Previous studies have demonstrated the feasibility of remote bonding for ultrasonic wave signal detection [19,20,21,22,23]. Wee [19] showed that remote bonding of FBG increases the amplitude of the output signal by a factor of 5 compared to direct bonding. Wu [20] reported that bonding the fiber optic at a distance from the FBG sensor can still measure ultrasonic waves and AE signals. Davis et al. [21] conducted experiments that showed differences between direct and remote bonding measurements. Yu et al. [22,23] combined the remote bonding method with high-sensitivity FBG sensors. Through experiments and finite element (FEM) analysis, their research also elucidated the mode conversion of AE waves from Lamb waves to longitudinal and transverse waves when entering cylindrical optical fibers through bonding points. FBG is more sensitive to longitudinal waves propagating in the fiber core than transverse wave modes appearing in the fiber’s outer diameter. Moreover, as longitudinal waves are dispersion-free in fiber-based ultrasonic waveguides, remote-bonded FBG can detect AE signals without distortion. However, the above research did not compare the changes in the damage signal received by FBG through remote bonding. This paper aims to locate the damage through remote bonding and study its changes. However, the above-mentioned research did not compare the changes in the signal received by FBG through remote bonding with and without damage, nor did it locate the damage through remote bonding. This paper aims to locate the damage through remote bonding. To further identify and locate damage, Sheen et al. [24] proposed a shape factor calculation method for evaluating the damage area in probability-based RAPID algorithm, which improved the imaging results. This paper will select different damage factors to improve the RAPID algorithm and apply the improved algorithm to image the damage and more accurately locate its position.

This paper is organized as follows: Firstly, the mechanism of Lamb waves in thin plate structures and the defect damage response mechanism for Lamb waves are introduced. Then, a remote bonding FBGs damage monitoring platform is established, and experiments are carried out to monitor the damage to aerospace aluminum alloy plates. Next, the analysis is conducted on enhancing the sensitivity of ultrasonic signals and the influence of bonding distance based on remote bonding. Finally, an improved RAPID is proposed to locate and image the hole defect.

## 2. Experiment

### 2.1. Experimental Platform Setup

**Figure 2:**
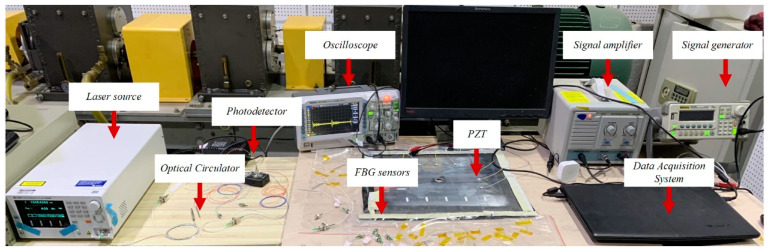
Damage monitoring system.

**Figure 3:**
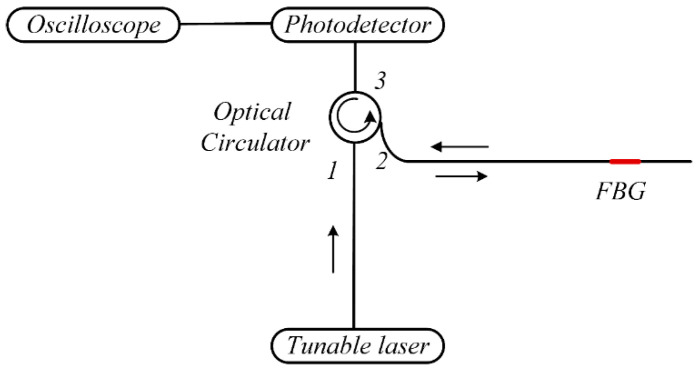
The schematic diagram of the demodulation system based on tunable laser source.

The UE-FBGs system(Sentec tsl-550, Komaki, Japan) built in this paper consists of two main components, as shown in Figure 2. One is the high-frequency demodulation system, which is mainly used for ultrasonic Lamb wave sensing and signal acquisition. It includes FBG sensors, an optical circulator, a laser source, a photodetector, and an oscilloscope. Figure 3 shows a schematic diagram of the wavelength demodulation system based on a tunable laser source. The narrow band light from the laser source enters the optical circulator from port 1 and is then transmitted from port 2 to the FBG. The light reflected from the FBG is then returned to port 2 and output from port 3 to the photodetector. The photodetector converts the light signal into an electrical signal, which is finally displayed by an oscilloscope.

The other is the ultrasonic excitation device, which is mainly used to generate ultrasonic excitation signal. In the experiment, the electrical signal is excited by the signal generator and transmits to the PZT exciter after passing through the signal amplifier. The PZT exciter converts the electrical signal into a mechanical vibration signal, thus generating a Lamb wave in the aluminum plate. The Lamb wave signal is received by the FBG transducer, demodulated by the high-frequency demodulation system and displayed on the digital oscilloscope.

**Figure 4:**
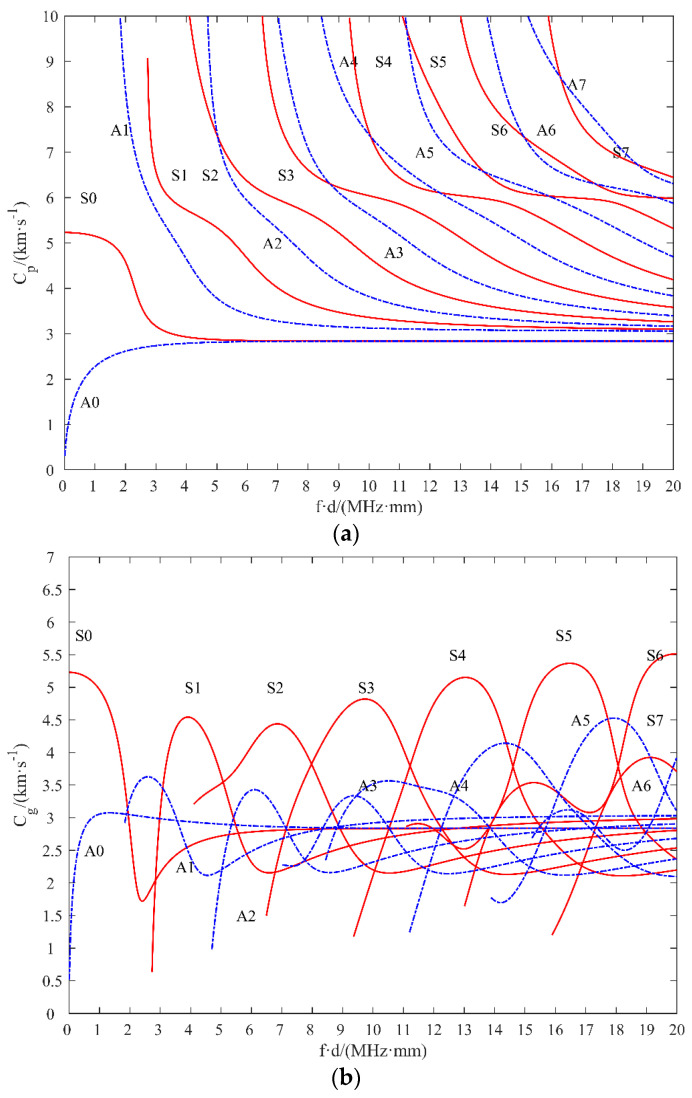
Dispersion curves for aluminum alloy plates at different frequency thickness products. (**a**) Phase velocity curves; (**b**) group velocity curves.

This paper applies Lamb wave-based SHM technology to the identification of hole defects in flat plate structures. Lamb waves are guided waves that propagate in thin plate structures and result from the superposition of longitudinal and transverse wave modes. During propagation, there are symmetric and asymmetric Lamb wave modes. The phase velocity of Lamb waves is a function of the product of the plate thickness and frequency. The dispersion curve of Lamb waves can be obtained by solving the Rayleigh–Lamb dispersion equation [25]. In this study, the 7075-series aluminum alloy plates are used with material parameters shown in Table 1 and the Chemical composition in Table 2, where the transverse wave velocity was 3050 m/s and the longitudinal wave velocity was 5928 m/s. The dispersion curves of the thin plate for different frequency and thickness products are calculated. As shown in Figure 4, only the zero-order Lamb wave exists when the frequency thickness product is less than 1 MHz × mm. Therefore, in order to obtain Lamb wave signals that are easier to analyze, a lower frequency thickness product is selected based on the dispersion curves to generate Lamb waves with single-order $A_{0}$ and $S_{0}$ modes.

### 2.2. Experimental Parameters Selection

The dispersion curve of Lamb waves indicates that the waveform pattern of Lamb waves is influenced by frequency. Additionally, the amplitude and phase of the Lamb wave waveform change with propagation distance. FBG exhibits a certain degree of directional sensitivity to Lamb waves [26]. Therefore, this study investigates the effects of FBG grating length, excitation frequency, and FBG placement on the response signal and selects appropriate parameters for experimentation.

#### 2.2.1. Grating Length

Under the UE, nonuniform strain will cause distortion in the reflection spectrum of the FBG, leading to adverse effects on demodulation. To reduce the impact of nonuniform strain on the detection results, it is necessary to determine the appropriate length of the FBG. Betz et al. [27] found through simulation that the wavelength of ultrasound should be at least 1.2 times the length of the FBG. Lee et al. [28] demonstrated experimentally that the FBG length must be less than half the wavelength of the ultrasound signal in order for the FBG sensor to have better reception performance for the excitation signal. In summary, the length of the FBG should be less than half of the wavelength. Based on the dispersion curve obtained, the frequency of the ultrasound should be less than 1 MHz, and the longitudinal wave velocity is 5928 m/s, corresponding to a wavelength of 5.9 mm. Half of the wavelength is approximately 3 mm. Therefore, an FBG sensor with a grating length of 3 mm was selected in this paper.

#### 2.2.2. Frequency

The experiment used a SMD10T2R111WL PZT exciter from Steaming Corporation (San Francisco, CA, USA), with a resonance frequency of 215 kHz. In order to make the generated Lamb wave signal have a higher amplitude, the center frequency of the signal generator should not deviate too much from the resonance frequency of the PZT exciter. Therefore, experiments were conducted at 15 different excitation frequencies, with a separation of 25 kHz between 100 and 450 kHz, to observe the effect of the excitation frequency on the FBG response signal. In the experiment, an FBG sensor with a grating length of 3 mm and a PZT exciter were glued to the surface of the plate using epoxy resin adhesive, and the distance between the FBG sensor and the PZT exciter was 200 mm.

**Figure 5:**
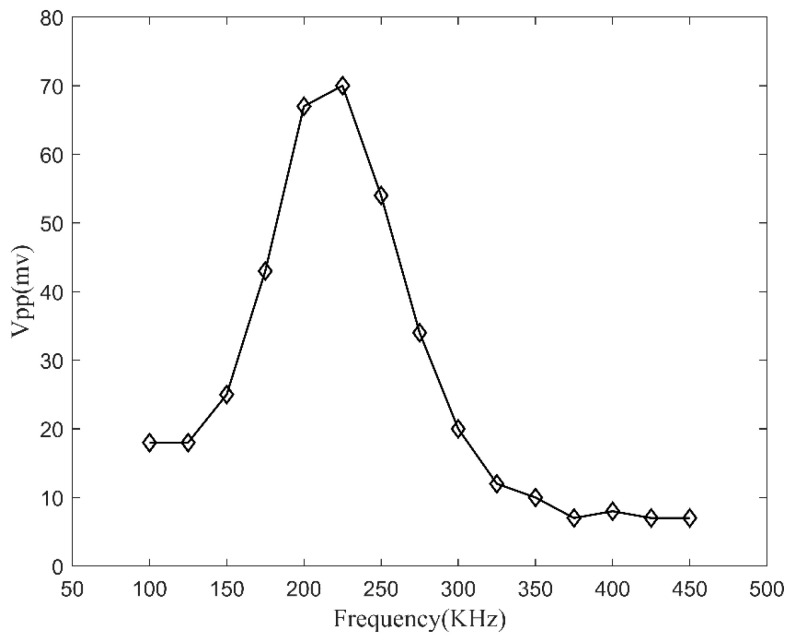
Variation of the amplitude of the FBG response signal at different excitation frequencies.

As shown in Figure 5, the FBG response *S*_0_ signal amplitude is low at lower frequencies and increases as the excitation frequency increases. When the excitation frequency reaches the resonance frequency of the piezoelectric element near 215 kHz, the FBG response signal amplitude reaches its maximum, about 70 mV. However, as the excitation frequency continues to increase beyond the resonance frequency of the piezoelectric element, the amplitude of the FBG response signal decreases. Therefore, the excitation frequency of the ultrasound should be close to the resonance frequency of the PZT element, and the excitation frequency can be set to 225 kHz.

### 2.3. Remote Bonding Parameters

In remote bonding configuration, the fiber acts as an ultrasonic waveguide to transmit ultrasonic waves from the bonding point to the FBG sensor [29]. In the case of direct bonding configuration, the shear hysteresis effect of the adhesive reduces the shear strain transmission along the length direction of the FBG structure, while the FBG in the remote bonding configuration is less sensitive to this effect [14]. In the remote bonding method, mode conversion and attenuation of ultrasonic waves occur during the propagation process from the fiber bonding point to the FBG sensor. In order to investigate the effect of the distance between the FBG and the bonding point on the response signal, appropriate remote bonding distances are selected to maximize the amplitude of the response signal, and experiments are conducted at different remote bonding distances.

**Figure 6:**
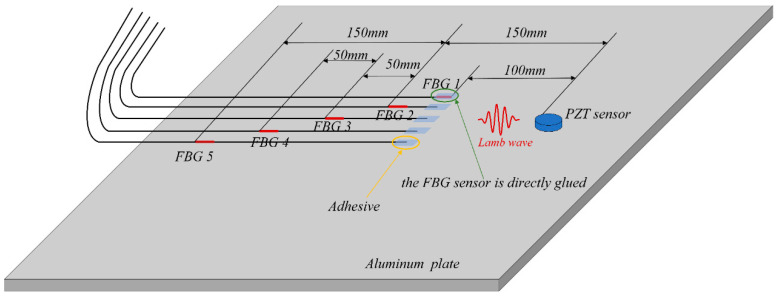
Diagram of FBG sensor remote attachment.

The excitation frequency of the experiment was set to 225 kHz, and the excitation signal and other settings were the same as above. A PZT exciter was glued to the surface of the plate, and five FBG sensors with a grating length of 3 mm were glued to the other edge of the aluminum plate, as shown in Figure 6. FBG1 was directly bonded, FBG2 was 50 mm away from the bonding point, FBG3 was 100 mm away, FBG4 was 150 mm away, and FBG5 was 200 mm away. The distance between the bonding point and the PZT exciter was 100 mm, and the distance between each FBG bonding point was controlled at 5 mm. Therefore, the propagation direction of the ultrasonic wave can be considered to be along the axial direction of the five fibers, and the effect of the incident angle of the ultrasonic wave on the signal can be neglected.

**Figure 7:**
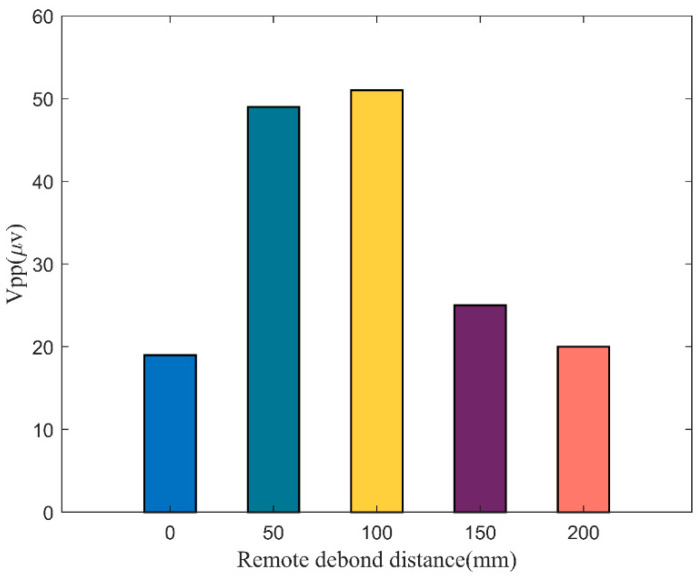
Peak-to-peak values at different locations for remote bonding.

The variation curves of the peak-to-peak values of the response signals of the FBG sensors bonded remotely are shown in Figure 7. It can be seen that the signal amplitudes of the four remote-bonded FBG are higher than that of the directly bonded, which verifies that remote bonding can effectively improve the sensitivity of the FBG in receiving ultrasonic signals. The signal amplitude of FBG2 is not significantly different from that of FBG3, indicating that the attenuation of the response signal is less severe when the remote bonding distance is shorter. However, when the remote bonding distance is further increased, the response signal suffers significant attenuation, and the signal amplitude decreases from 51 mV to about 20 mV.

**Figure 8:**
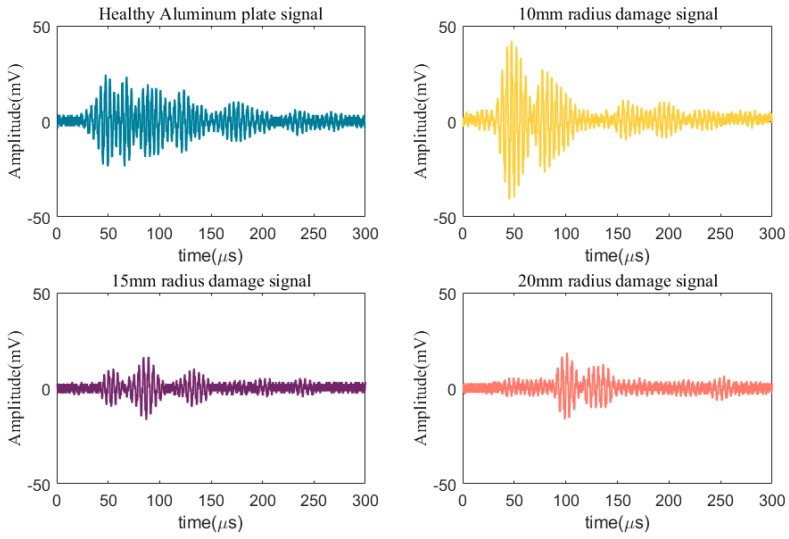
Lamb wave signals received at different remote bonding distances.

Figure 8 shows the Lamb wave signals received under different remote bonding distances. It can be observed that when the remote bonding distance is too short, the S_0_ and A_0_ modes of the Lamb wave signal are not completely separated, which makes it difficult to extract the S_0_ mode signal for analysis. At a remote bonding distance of 50 mm, the S_0_ and A_0_ modes still overlap with each other. As the remote bonding distance increases, the S_0_ and A_0_ modes gradually separate and form complete and separate wave packets. Therefore, this study uses a remote bonding distance of 100 mm, which results in less signal attenuation and the S_0_ and *A*_0_ modes are fully separated and thus facilitate analysis.

### 2.4. Experimental Setup

**Figure 9:**
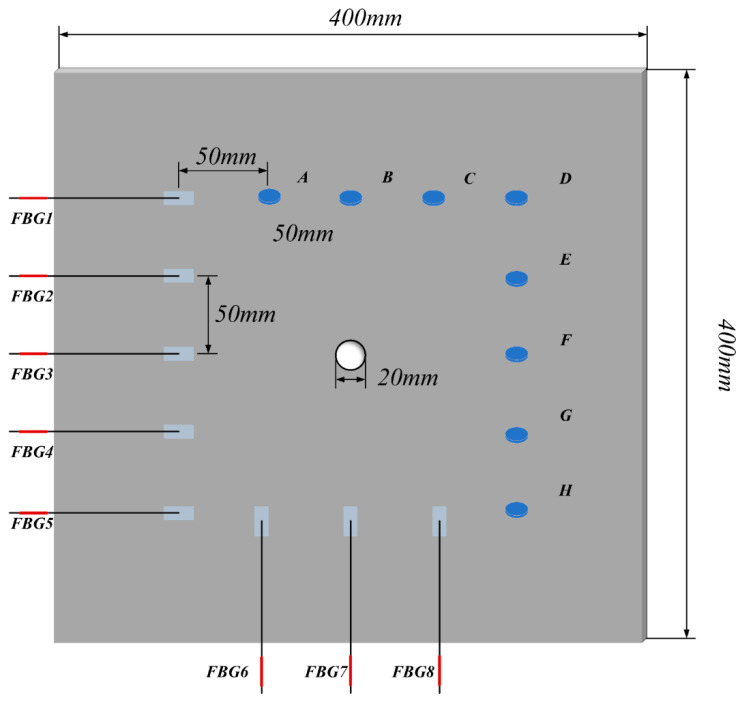
Schematic layout of exciters and sensors.

The test specimens used in the experiment were 7075 aluminum alloy plates with dimensions of 400 mm × 400 mm × 1 mm. The sensors layout is shown in Figure 9, which consists of 8 PZT exciters and 8 FBG sensors. The PZT exciters were numbered A to H, with a spacing of 50 mm between two adjacent exciters. The spacing between the 8 FBG sensors was also 50 mm, with a remote debonding distance of 100 mm. They were numbered 1 to 8 and were used to monitor a square area with a side length of 200 mm. The study simulated damage using a hole defect located at the center of the monitored area with a diameter of 20 mm. In the experiment, FBG sensor grating length was 3 mm. The excitation signal was a 5-cycle sine wave modulated by a Hanning window, with an amplitude of 5 V generated by a signal generator in burst mode. After amplification by a signal amplifier, the amplitude of the excitation signal was 110 V. The signal was applied separately with each piezoelectric exciter and the frequency was 225 KHz. The tunable laser light source had an optical power of 6 dBm, and the gain of the photodetector was set to 30 dB.

## 3. Results

**Figure 10:**
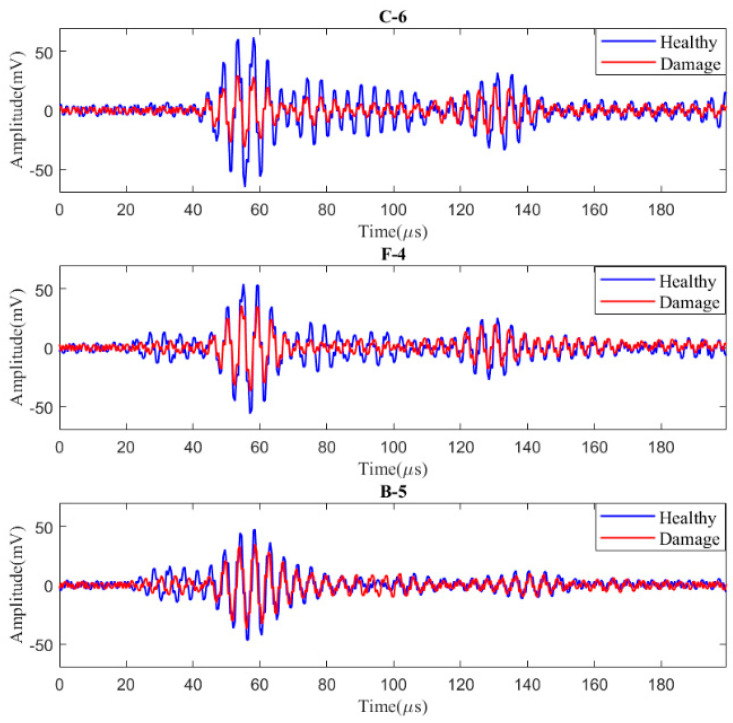
Experimental signals for different pathways with and without damage.

Based on the experimental results, the signals from different sensing paths in intact and damaged states are collected for comparison and analysis. Paths C-6, F-4, and B-5 are analyzed as examples, and the extracted *S*_0_ mode wave packets are shown in Figure 10. Among them, path C-6 directly passes through the damage, and the extracted *S*_0_ mode signal shows a significant reduction in amplitude. Path F-4 is relatively close to the damage and is also affected to a certain extent. Path B-5 is the farthest from the hole, and its *S*_0_ mode signal remains almost unchanged with and without the hole damage.

Furthermore, an imaging algorithm is required to determine the damage location. The damage localization and visualizing mainly involve three parts: First, signal processing, which applies wavelet denoising methods to preprocess the signals. Second, the sensitivity of the signals to various damage indexes (DI) is analyzed. Third, with DI as input, damage localization and imaging are achieved through an improved reconstruction algorithm for probabilistic inspection of damage (RAPID) [30].

### 3.1. Signal Processing

The Lamb wave signals received by the FBG sensor always have low amplitude and are susceptible to environmental noise interference in practice. It can cause false alarms or missed detections, which will degrade the accuracy and reliability of structural damage monitoring. Therefore, signal processing methods need to be adopted to reduce noise in Lamb wave signals and extract effective information related to damage. The widely used Lamb wave signal denoising methods include wavelet denoising [31] and empirical mode decomposition [32]. Compared to the latter, wavelet transform has better time-frequency localization characteristics, which can preserve local signal features and reduce noise well. Therefore, wavelet transform was selected to denoise the experimentally acquired signals.

#### 3.1.1. Lamb Wave Signal Denoising

The wavelet denoising consists of the following processes: decomposition process, a wavelet is selected to perform n-level wavelet decomposition on the signal; thresholding process, the coefficients of each level of the decomposition are thresholded to obtain the estimated wavelet coefficients; reconstruction process, wavelet reconstruction is performed according to the denoised wavelet coefficients to obtain the denoised signal.

There are three key problems that need to be solved in this process: determining a suitable threshold and selecting a threshold function, selecting the wavelet basis function, and selecting the wavelet basis order and decomposition level.

(1) Selection of wavelet threshold and threshold function

The selection of threshold and threshold function has a significant impact on the denoising. The widely used threshold functions can be classified into soft and hard threshold functions. The hard threshold function has a discontinuity problem, and its reconstructed signal has a higher mean square error and a less effective denoising performance than the soft threshold function. Therefore, the soft thresholding function is adopted to denoise Lamb wave signals in our investigation [33].

The noise variance σ is as follows:

(1) $$\sigma =\frac{median(\left|d_{1}\right|)}{0.6745}$$

The generic soft threshold *l* is as follows:

(2) $$l=\sigma \sqrt{2\mathrm{In}N}$$

where, $d_{1}$ is the wavelet transform coefficient and *N* is the signal length.

The expression for the soft thresholding function is:

(3) $$d_{jh}=\{\begin{array}{c} \mathrm{sgn}(d_{j})(\left|d_{j}\right|-l),\\ 0 \end{array}\begin{array}{c} \left|d_{j}\right|\geq l\\ \left|d_{j}\right|<l \end{array}$$

where sgn() is the sign function, *d_jh_* is the thresholder wavelet transform coefficient, and *j* is the decomposition scale. After thresholding the wavelet coefficients, the denoised Lamb wave signal can be obtained by signal reconstruction.

(2) Selection of wavelet basis function, wavelet basis order and decomposition level

There are multiple types of basis functions in wavelet analysis, and the different basis functions usually lead to different results. Therefore, it is necessary to select suitable wavelet basis functions to achieve the best denoising performance. Generally, the orthogonal, symmetrical, and smooth wavelet basis functions are selected, such as Daubechies (*dbN*) wavelet basis function, Symlet (*symN*) wavelet basis function, and Coiflet (*coifN*) wavelet basis function. This article selects these three wavelet basis functions for denoising processing and comparative analysis.

Besides the wavelet basis function, the order and decomposition level have significant impacts on the denoising performance as well. Currently, there are no unified methods for selecting the most suitable order and decomposition level. In this study, the appropriate parameters are determined empirically through the simulation experiments.

#### 3.1.2. Evaluation Metrics for Denoising Performance

Two commonly used metrics, *SNR* and root-mean-square error (*RMSE*), are employed herein to evaluate the denoising performance.

The formula for calculating SNR is:

(4) $$SNR=10\;\log_{10}\frac{\sum_{n=1}^{N}x{\left(n\right)}^{2}}{\sum_{n=1}^{N}{\left[x^{\prime }(n)-x(n)\right]}^{2}}$$

The *RMSE* is calculated as:

(5) $$RMSE=\sqrt{\frac{1}{N}\sum_{n=1}^{N}{\left[x^{\prime }(n)-x(n)\right]}^{2}}$$

where *x* (*n*) represents the original signal, $x^{\prime }(n)$ represents the denoised signal, *N* represents the noise present in the original signal.

#### 3.1.3. Simulation Experiment for Denoising

**Figure 11:**
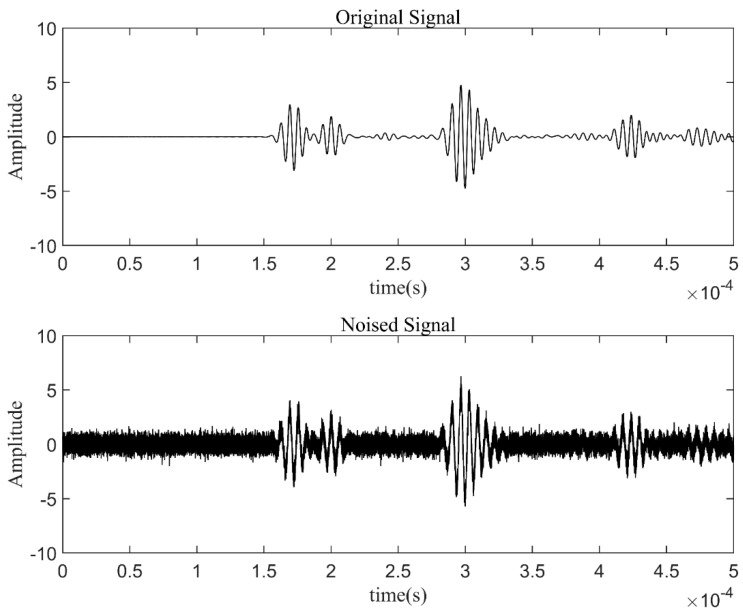
Simulated signals.

Finite element simulation (FEM) was performed for the Lamb wave signal of a complete plate. A three-dimensional finite element analysis was conducted in the ABAQUS/CAE2022 software environment. An aluminum plate with a geometric size of 400 mm × 400 mm × 1 mm was considered, as shown in Figure 9. The simulation specimen’s material parameters, sensor configuration, and excitation signal settings were the same as those in the experiment. The excitation signal was a 225 kHz 5-cycle sinusoidal tone burst modulated by a Hanning window, which was generated by applying the dynamic concentrated force to the PZTA model. The corresponding average element size was 0.001 m. Then, the simulation signal was received through the PZTA-FBG8 channel. The Gaussian white noise with a SNR of 5 dB was added to the simulation signal to obtain the noisy signal, as shown in Figure 11.

**Figure 12:**
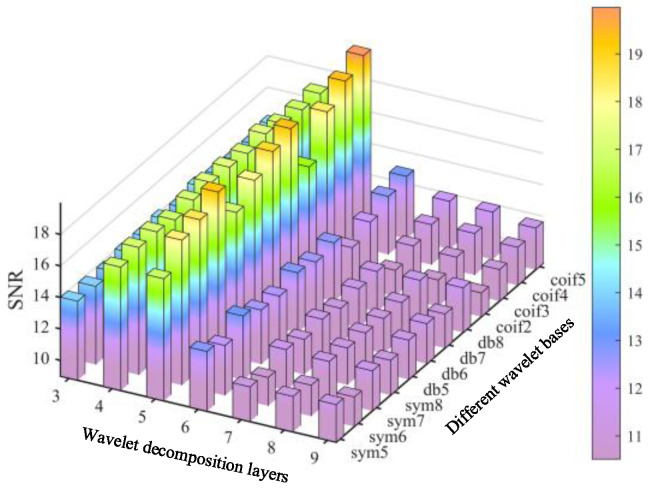
SNR for different wavelet bases and decomposition levels.

In this study, three types of wavelet bases, namely Daubechies (*dbN*), Symlet (*symN*), and Coiflet (*coifN*), were selected for the denoising simulation experiment. For each basis function, a two-parameter orthogonal experiment was conducted. The order of the *db* and *sym* wavelet basis ranged from 5 to 8, and the decomposition level ranged from 3 to 9. Due to the order of the *coif*, wavelet basis can not be greater than 5, it was set to 2 to 5, and the decomposition level was 3 to 9. Figure 12 shows the SNR value variation for the different wavelet bases, orders and decomposition levels. With the decomposition level increasing, the value of SNR first increased and then decreased for all of the given conditions. Larger SNR value indicates better denoising result, so the optimal decomposition level for each basis function was 5 in this investigation. Then, after comparing the different basis functions and the corresponding orders, it was clear that the maximum SNR value appeared at the *coif* wavelet and 5 order.

**Figure 13:**
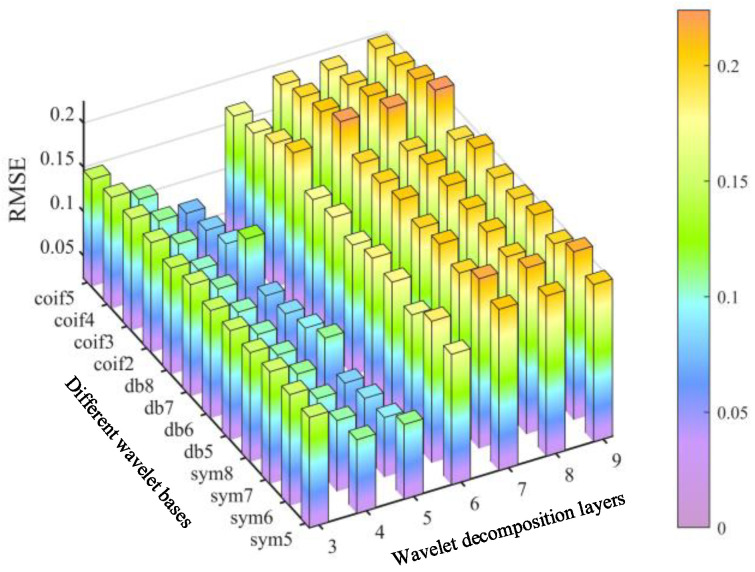
RMSE for different wavelet bases and decomposition levels.

Similarly, the RMSE’s of the denoised signal under the different wavelet bases, orders and decomposition levels are plotted in Figure 13. Unlike the SNR, the smaller RMSE value indicates the better denoising performance. It is clear that the optimal decomposition level for each basis function is also 5. And the RMSE of the 5-order *coif* function reaches the minimum value, as shown in Table 3.

**Figure 14:**
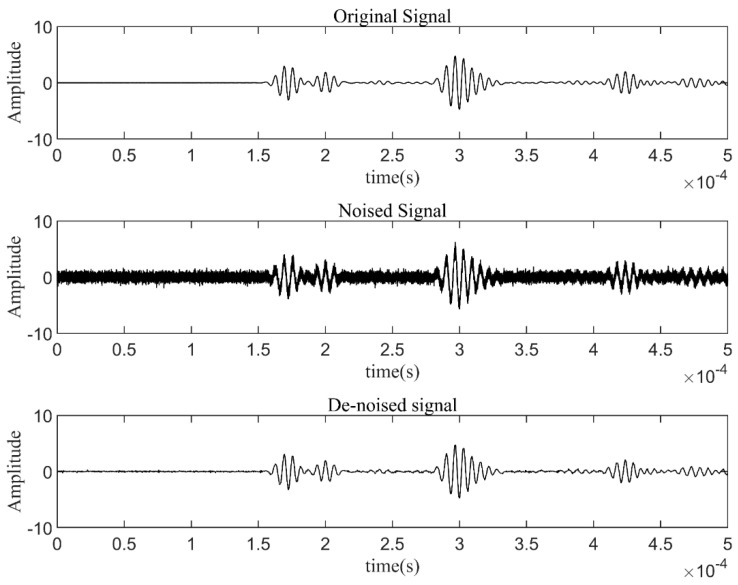
Original signal, noised signal, and signal denoised by coif wavelet.

Using the above optimized parameters, the denoising comparison results are shown in Figure 14, respectively, including original signal, noised signal, and signal denoised by *coif* wavelet. It can be seen the denoised signal is very close to the original one, whose SNR and RMSE are 19.9712 dB and 0.0754, respectively. Therefore, the *coif* wavelet basis with an order of 5 and a denoising decomposition level of 5 was selected to denoise the Lamb wave signals in this study.

### 3.2. Damage Indexes

The Lamb wave-based damage monitoring technique determines the damage mainly based on the damage indexes (*DI*), which characterize the signal variation with and without the damage impact. In this paper, the root-mean-square error (*RMSE*) [34], signal difference coefficient (*SDC*) [6], damage index based on signal energy [35], and normalized correlation moment (*NCM*) [36] are employed as the *DI*.

(1) *RMSE*

(6) $$RMSE=\sqrt{\frac{1}{N}\sum_{n=1}^{N}{\left[x(n)-y(n)\right]}^{2}}$$

where, *x* (*n*) is the reference signal under undamaged conditions, *y* (*n*) is the real-time collected damaged signal, *N* is the length of the signal.

(2) *SDC*

(7) $$SDC=1-\left|\frac{\sum_{n=1}^{N}\left[x(n)-\bar{x}][y(n)-\bar{y}\right]}{\sqrt{\sum_{n=1}^{N}{\left[x(n)-\bar{x}\right]}^{2}\sum_{n=1}^{N}{\left[y(n)-\bar{y}\right]}^{2}}}\right|$$

where, $\bar{x}$ is the mean value of the reference signal, $\bar{y}$ is the mean value of the damaged signal.

(3) $E_{3}$

(8) $$E_{3}=\frac{\sum_{n=1}^{N}{\left|x(n)\right|}^{2}-\sum_{n=1}^{N}{\left|y(n)\right|}^{2}}{\sum_{n=1}^{N}{\left|y(n)\right|}^{2}}$$

(4) *NCM*

(9) $$NCM=\frac{\sum_{n=1}^{N}n^{k}\left|r_{xy}(n)\right|-\sum_{n=1}^{N}n^{k}\left|r_{xx}(n)\right|}{\sum_{n=1}^{N}n^{k}\left|r_{xx}(n)\right|}$$

where $r_{xy}(n)$ is the cross-correlation function between the reference signal and the damaged signal, $r_{xx}(n)$ is the autocorrelation function of the reference signal, and its expression is:

(10) $$r_{xy}(n)=\sum_{n=1}^{N}x(n)y(N-n)$$

(11) $$r_{xx}(n)=\sum_{n=1}^{N}x(n)x(N-n)$$

where *k* is the order of the statistical moment. It is found that the damage sensitivity reaches the highest level when *k* is set to 0.01 [36].

**Figure 15:**
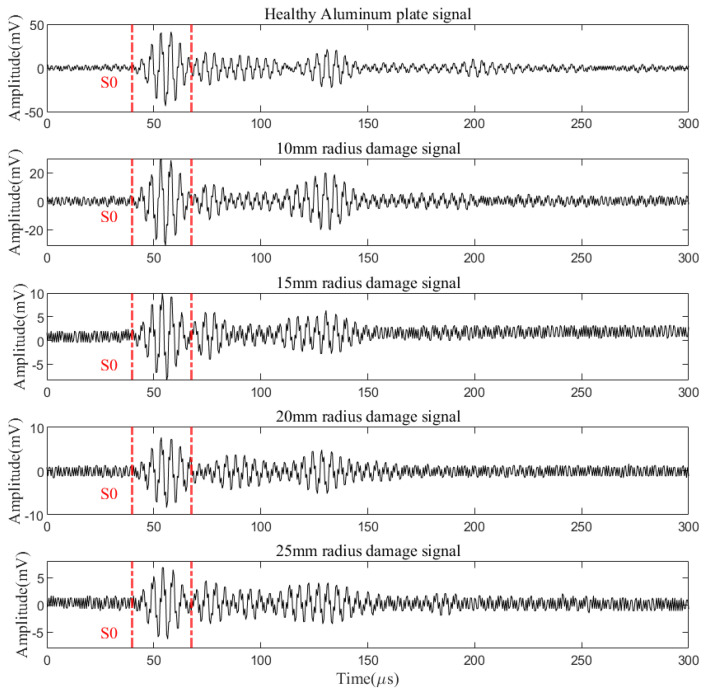
Lamb wave signal of the FBG8 sensor signal at different levels of damage.

To compare the sensitivity of different DI, experiments are conducted, in which the specimen configurations are shown in Figure 15. The radii of the hole damage are set to 10, 15, 20, and 25 mm, respectively. The PZTA generates the exciting signal and the FBG8 sensor is used to receive the signal. The received signals for different damage sizes are plotted in Figure 15.

It is clear that the amplitude of the damage signal is significantly smaller than the original one. And the amplitude is negatively correlated with the damage radius.

**Figure 16:**
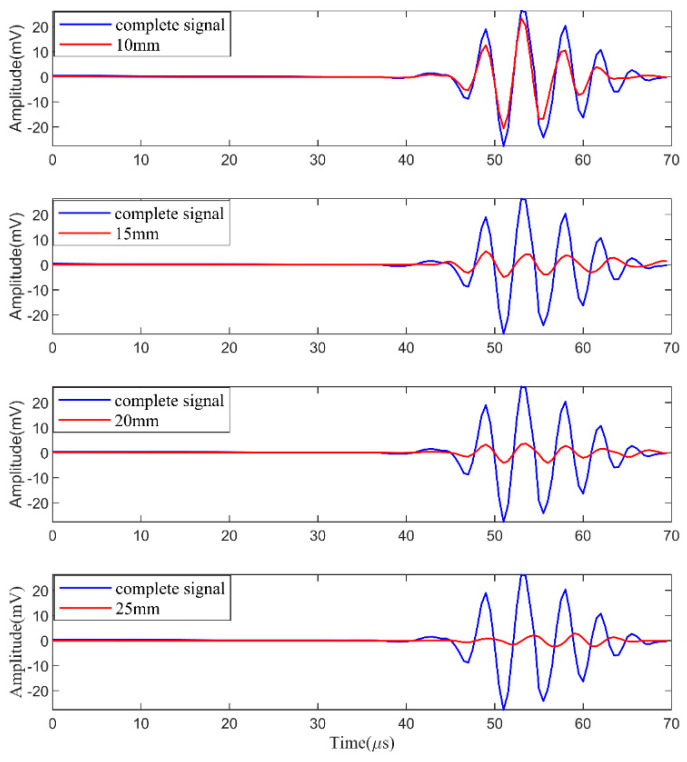
Comparison signals after denoising.

Because of the higher sensitivity of FBG sensors to the *S*_0_ mode wave packet than to the *A*_0_ mode, the *S*_0_ mode wave packet is extracted for signal analysis. The *coif*5 wavelet is used to denoise. The final extracted *S*_0_ mode wave packets at different degrees of damage are shown in Figure 16. After denoising, the *S*_0_ mode wave packets become smooth and easy to analyze.

**Figure 17:**
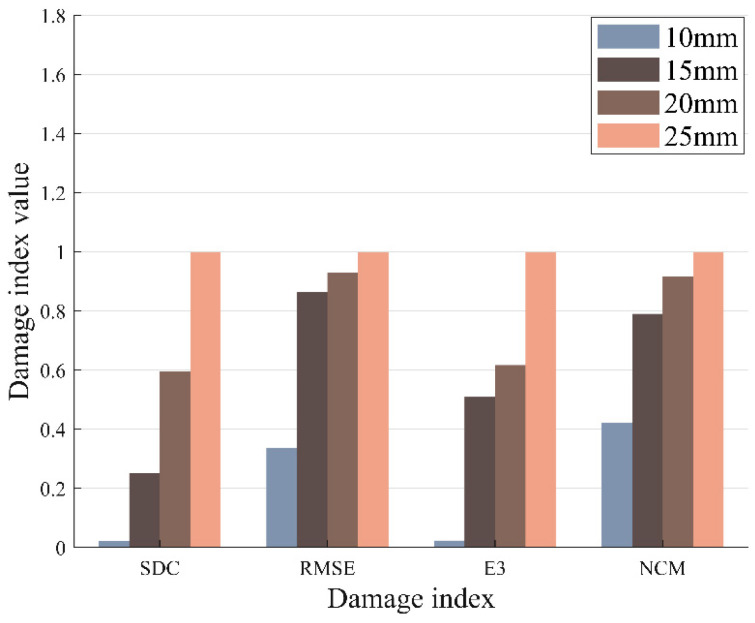
The different damage indexes vs. the normalized damage value.

The values of the different DI at different damage levels can be calculated using the extracted *S*_0_ mode packets. After normalization, the variation of different DI with increasing damage levels is demonstrated in Figure 17. It can be seen that *SDC*, *RMSE*, *E*_3_, and *NCM* increase with the extension of the damage radius. *SDC* and *E*_3_ are not sensitive to the small damage. Moreover, all DI exhibit a positive correlation with the degree of structural damage.

### 3.3. The Improved Imaging Algorithm

**Figure 18:**
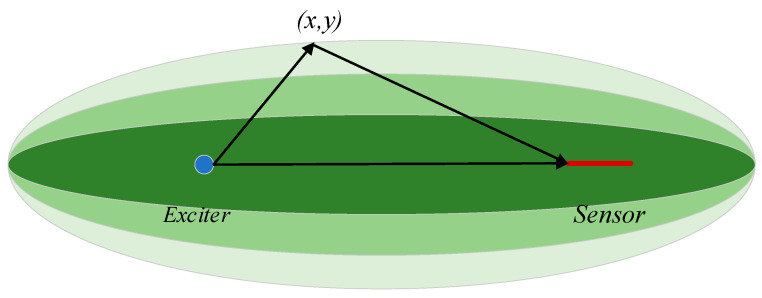
The spatial distribution probability of the DI. Blue dot—PZT Exciter; Red arrow—FBG Sensor.

The main principle of the RAPID imaging algorithm is as follows: firstly, the *DI* value is calculated for each excitation–sensor path (including *SDC*, *RMSE*, *E_3_*, and *NCM*), which is used to compute the damage probability. Then, the *DI* values along the excitation–sensor paths are allocated to the regions around the paths, typically by constructing a spatial probability distribution function. The contour map of the spatial probability distribution function is elliptical, as shown in Figure 18. The excitation *i* and sensor *j* are the two foci of the ellipse, and the long axis of the ellipse lies on the excitation–sensor path. The damage probability value at any point within the ellipse decreases as the sum of its distances to the two foci increases, and it equals zero when that distance gets a given large value.

The spatial probability distribution function is defined as follows:

(12) $$\{\begin{array}{l} s_{ij}(x,y)=\frac{\beta -R_{ij}(x,y)}{\beta -1},\beta >R_{ij}\\ s_{ij}(x,y)=0,\;\;\;\;\;\;\;\;\;\;\;\;\;\beta \leq R_{ij}\; \end{array}$$

where *S_ij_* represents the spatial distribution probability of the DI values at any point within the monitoring area, and the parameter *β* controls the size of the ellipse. The shape parameter *β* needs to be greater than 1. $R_{ij}(x,y)$ is the ratio between the sum of the distances from point (*x*, *y*) to the exciter $\left(x_{ik},y_{ik}\right)$ and sensor $\left(x_{jk},y_{jk}\right)$ and the double focal length, and it can be expressed as:

(13) $$R_{ij}=\frac{\sqrt{{\left(x-x_{ik}\right)}^{2}+{\left(y-y_{ik}\right)}^{2}}+\sqrt{{\left(x-x_{jk}\right)}^{2}+{\left(y-y_{jk}\right)}^{2}}}{\sqrt{{\left(x_{ik}-x_{jk}\right)}^{2}+{\left(y_{ik}-y_{jk}\right)}^{2}}}$$

Finally, to obtain the image of damage probability distribution, all the ${DI}_{ij}$ on the exciter–receiver paths are multiplied by the approximate distribution $s_{ij}\left(x,y\right)$ and summed up. The probability of the existence of damage at any point (*x*, *y*) in the detection area is:

(14) $$P(x,y)=\sum_{i=1}^{N}\sum_{j=1}^{N}s_{ij}(x,y)\times D_{ij}$$

where *N* represents the number of exciter–sensor paths.

**Figure 19:**
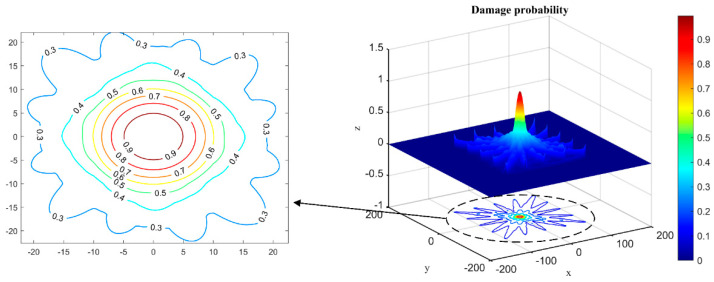
The damage probability image and contour map.

The 20 mm diameter hole damage is localized and visualized by using the experimental setup in Section 2.4. The *S*_0_ mode wave packet is extracted from the real-time damage signal and the original signal, and the *DI* values are calculated for each path. After that, the probability of damage is calculated and normalized. Figure 19 shows the damage probability image and contour map of *NCM*. It is obvious that the gradient changes uniformly when the probability is larger than 0.6. Therefore, the threshold for predicting the probability of damage can be set to 0.6.

**Figure 20:**
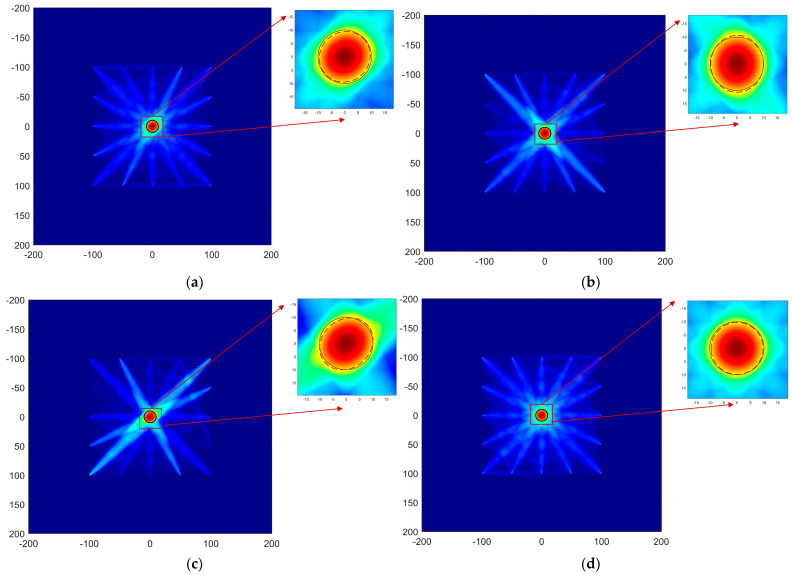
Image of different damage indices for imaging performance. (**a**) SDC; (**b**) RMSE; (**c**) E_3_; (**d**) NCM.

The damage probability images are shown in Figure 20. The blue areas in the figure represent low damage probability, while the red areas indicate high damage probability. The black circle is the actual damage location and the red circle is the prediction. It can be seen that the imaging results using *SDC*, *RMSE*, *E3*, and *NCM* are all good. Moreover, the predicted damage shape by *NCM* is slightly closer to the actual shape than the other DI value.

The predicted damage areas for each DI are calculated pixel-by-pixel within the threshold, as shown in Table 4. The imaging results by using *RMSE* and *NCM* are better than the conventional method with *SDC*, with errors smaller than 2%.

## 4. Discussion and Conclusions

In this paper, the damage monitoring investigation based on the remote bonding fiber Bragg grating sensing is performed. Predictive damage imaging has a satisfactory effect. Based on the current study, the following conclusions can be drawn:

(1) A remote bonding FBGs damage monitoring system is established. The signal response characteristics of direct bonding and different remote bonding distances are investigated. It is found that 100 mm is the appropriate remote bonding distance, which ensures small signal amplitude attenuation and is convenient for signal extraction and analysis.
(2) The soft threshold wavelet transform-based noise reduction is investigated. Different wavelet basis functions, orders and decomposition levels have an impact on the noise reduction results, and the *coif*5 wavelet with five decomposition levels has the best effect. A sensitivity analysis of the damage index is performed, which reveals that *SDC*, *RMSE*, *E3*, and *NCM* are all sensitive enough to give an accurate prediction of the location and shape of the damage.
(3) The damage localization and imaging of hole defects is achieved by an improved damage probability detection reconstruction algorithm with an error smaller than 2%.

The damage monitoring study will be extended to more complex structures and different shapes of damage in the future.

## Figures and Tables

**Table 1:** Aluminum alloy material parameters.

Elastic Modulus (*E*)	Poisson Ratio (*v*)	Density (*ρ*)
70 GPa	0.32	2.85 g/cm^3^

**Table 2:** Chemical composition of 7075 aluminum alloy.

	Si	Fe	Cu	Mn	Mg	C	Zn	Ti	V	Zr
Max % weight	0.4	0.50	2.0	0.30	2.9	0.28	6.1	0.20	0.05	0.05

**Table 3:** RMSE for different wavelet bases at decomposition level 5.

Wavelet Function Name	*sym*8	*db*8	*coif*5
RMSE	0.0817	0.0813	0.0754

**Table 4:** Monitor structural hole defect imaging accuracy.

DI	Damage Area/mm^2^	Predicted Area/mm^2^	Damage Shape Ratio %
*SDC*	314	296.75	94.50
*RMSE*	314	317.25	101.03
*E_3_*	314	298	94.90
*NCM*	314	307.75	98.01

## Data Availability

Data will be made available on request.
